# TBL1 is required for the mesenchymal phenotype of transformed breast cancer cells

**DOI:** 10.1038/s41419-019-1310-1

**Published:** 2019-01-31

**Authors:** Sabrina Rivero, Elena Gómez-Marín, José A. Guerrero-Martínez, Jorge García-Martínez, José C. Reyes

**Affiliations:** Centro Andaluz de Biología Molecular y Medicina Regenerativa-CABIMER, Consejo Superior de Investigaciones Científicas-Universidad de Sevilla-Universidad Pablo de Olavide, Av. Americo Vespucio, 41092 Seville, Spain

## Abstract

The epithelial-to-mesenchymal transition (EMT) and its reversion (MET) are related to tumor cell dissemination and migration, tumor circulating cell generation, cancer stem cells, chemoresistance, and metastasis formation. To identify chromatin and epigenetic factors possibly involved in the process of EMT, we compare the levels of expression of epigenetic genes in a transformed human breast epithelial cell line (HMEC-RAS) versus a stable clone of the same cell line expressing the EMT master regulator ZEB1 (HMEC-RAS-ZEB1). One of the factors strongly induced in the HMEC-RAS-ZEB1 cells was Transducin beta-like 1 (TBL1), a component of the NCoR complex, which has both corepressor and coactivator activities. We show that TBL1 interacts with ZEB1 and that both factors cooperate to repress the promoter of the epithelial gene E-cadherin (*CDH1*) and to autoactivate the *ZEB1* promoter. Consistent with its central role, TBL1 is required for mesenchymal phenotypes of transformed breast epithelial and breast cancer cell lines of the claudin-low subtype. Importantly, a high expression of the *TBL1* gene correlates with poor prognosis and increased proportion of metastasis in breast cancer patients, indicating that the level of TBL1 expression can be used as a prognostic marker.

## Introduction

Epithelial and mesenchymal cellular phenotypes are the edges of a spectrum of states that can be transitory or stable^1^. The process by which epithelial cells can downregulate epithelial characteristics and acquire a mesenchymal phenotype is called epithelial-to-mesenchymal transition (EMT) and the reverse process, mesenchymal-to-epithelial transition (MET). Both processes are not only common during embryonic development^2^ but are also involved in different stages of the metastatic cascade, including tumor cell dissemination and migration^3^, generation of tumor circulating cells^4^, cancer stem cells^5,6^, chemoresistance^7,8^, and metastasis formation^9–12^. During EMT, cells undergo an extensive reorganization of cell junction complexes, cytoskeletal architecture, and extracellular matrix interactions^1,2,13^. Further, cells increase their motility and invasion properties and become more resistant to drugs. These transformations require large changes in gene expression, which are controlled by master transcription factors (EMT-TF), including SNAIL (SNAI1 and SNAI2), TWIST, and zinc-finger E-box-binding (ZEB) transcription factors (ZEB1 and ZEB2)^13^. The SNAI1 and ZEB proteins are repressors of epithelial genes and activators of mesenchymal genes. Multiple signaling pathways, including transforming growth factor (TGF)-β, WNT, Notch, and mitogen-activated protein kinases, cooperate (in either an autocrine or paracrine manner) to initiate EMT by increasing EMT-TF expression^13^.

Both EMT and MET require extensive reorganization of the epigenetic information of the cells^14,15^. For example, SNAI1 represses transcription of epithelial genes, such as *CDH1* (which encodes E-cadherin), by recruiting chromatin-modifying machineries, including the Polycomb repressive complex 2, the Lys-specific demethylase 1/REST corepressor 1 complex, and H3K9 histone methyltransferases^16–19^. ZEB1 has been also shown to repress *CDH1* by recruiting the corepressor CtBP1^20^ and the chromatin remodeler BRG1^21^. Thus identifying epigenetic and chromatin regulators involved specifically in EMT and MET is of paramount importance for better understanding the mechanisms responsible for tumor cell dissemination and metastasis formation, as well as for identifying putative druggable targets. With this purpose, we analyzed previously published expression data of a RAS-transformed human mammary epithelial cell line (HMEC-RAS) versus a stable clone of the same cell line expressing ZEB1 and with a strong mesenchymal phenotype (HMEC-RAS-ZEB1)^22^. We rationalized that epigenetic genes strongly upregulated in the ZEB1 expressing cells may be essential for the mesenchymal phenotype. One of the most upregulated genes was Transducin beta-like 1 (*TBL1*), encoding a transcriptional cofactor that was described as a component of the nuclear receptor corepressor (NCoR)/silencing mediator for retinoid and thyroid receptors (SMRT)/histone deacetylase 3 complex^23^. TBL1 overexpression has been linked to pancreas cancer^24^ and oncogenic Wnt signaling^25^, but its role in controlling the mesenchymal phenotype has not been analyzed. We show that TBL1 interacts and cooperates with ZEB1 for repressing the *CDH1* promoter and for self-activation of the *ZEB1* promoter and that it is essential for the mesenchymal and stem-like phenotypes. Downregulation of TBL1 in breast cancer cell lines decreased cell invasion ability. In agreement with this, human breast cancer tumors with high expression of the *TBL1* gene correlates with poor prognosis and an increased proportion of metastasis.

## Results

### Differential expression of epigenetic genes in epithelial versus mesenchymal mammary cells

**Fig. 1:**
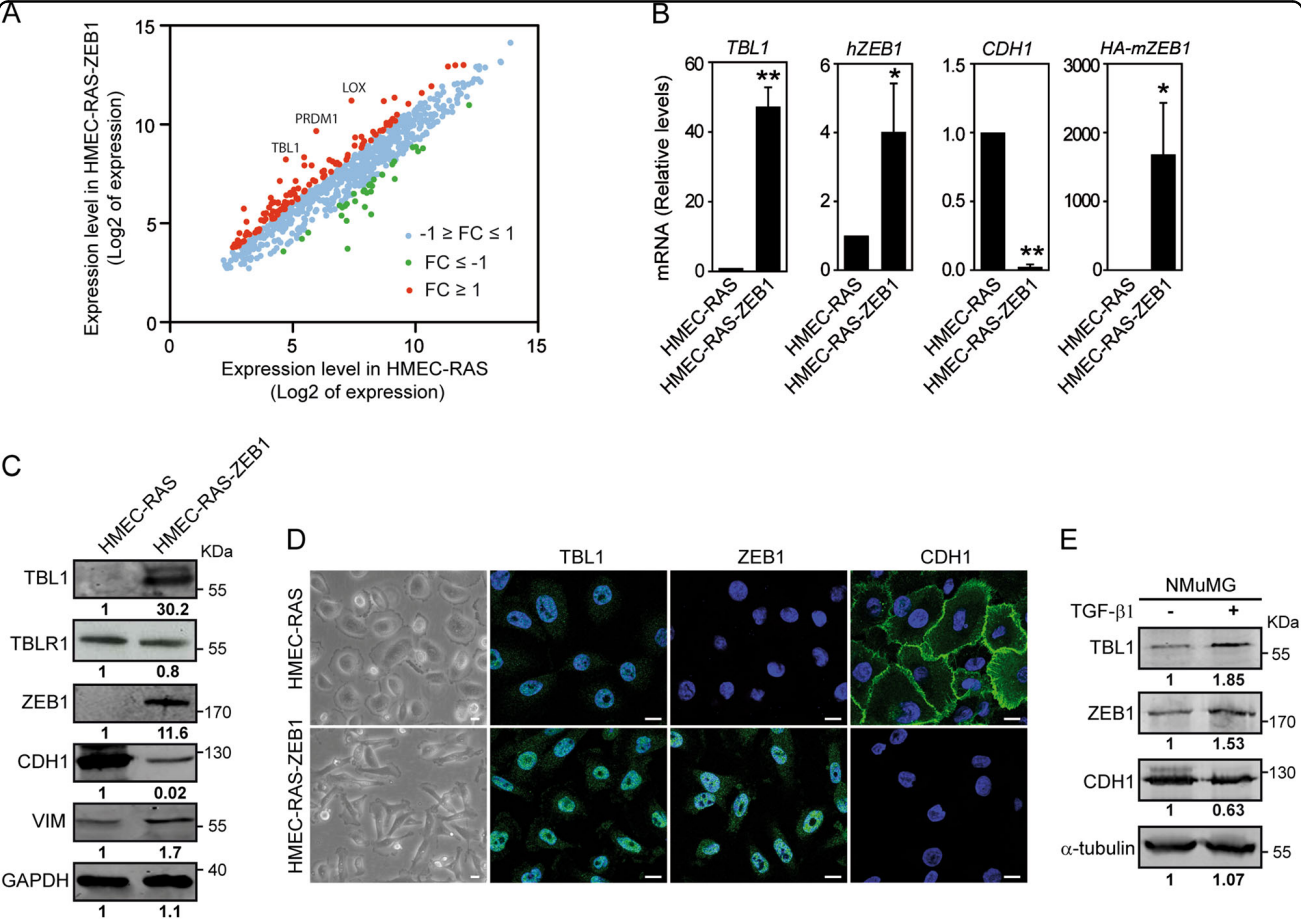
Differential expression of epigenetic genes in epithelial versus mesenchymal mammary cells. **a** Scatter plot showing expression profiles (Log_2_ scale) of 824 genes encoding epigenetic and chromatin factors (supplementary Table S1) from HMEC-RAS cells (*x* axis) and HMEC-RAS-ZEB1 cells (*y* axis). Data obtained from GEO accession GSE32727^22^. Genes upregulated or downregulated more than two-fold (Log_2_ (fold change) ≥ 1 or Log_2_ (fold change) ≤ 1) are depicted in red or green, respectively. **b** *TBL1*, *hZEB1*, *CDH1*, and *mZEB1* mRNA levels were determined by reverse transcription–quantitative real-time polymerase chain reaction in HMEC-RAS and HMEC-RAS-ZEB1 cells. Data are the average of *n* = 6 data from three independent experiments ± SD. *p < 0.05, **p < 0.01 versus control by ANOVA. **c** Whole-cell extracts from HMEC-RAS and HMEC-RAS-ZEB1 cells were analyzed by western blot with the indicated antibodies. The anti-ZEB1 antibody recognizes both proteins, the endogenous hZEB1 and the transgenic HA-mZEB1. Quantification of different signals is shown. **d** Immunofluorescence staining to detect the presence of the indicated proteins in HMEC-RAS and HMEC-RAS-ZEB1 cells. Nuclei were stained with 4,6-diamidino-2-phenylindole. Bar, 10 μm. **e** Western blot analysis of TBL1, ZEB1, CDH1, and α-tubulin with proteins extracted from NMuMG cells untreated and treated with 10 ng/ml TGF-β1 for 48 h. Quantification of different signals is shown

To determine EMT-dependent changes of gene expression of a set of 824 known and predicted chromatin and epigenetic factors (Supplementary Table S1), we analyzed previously published expression data of a H-RAS^G12V^-transformed human mammary epithelial cell line (HMEC-RAS) versus a stable clone of the same cell line expressing a recombinant mouse HA-tagged *ZEB1* (HA-*mZEB1*) gene (HMEC-RAS-ZEB1)^22^. HMEC-RAS-ZEB1 cells exhibit the transcriptional signature of claudin-low tumors, including low expression of tight and adherens junction genes, mesenchymal traits, and stem cell-like characteristics^22^. Out of the 824 chromatin and epigenetic genes, 112 were upregulated (Log_2_ fold-change (FC) ≥ 1) and 32 were downregulated (Log_2_ FC ≤ −1) (Supplementary Table S1 and Fig. 1a). The two most upregulated gene were *LOX* and *PRDM1* (*BLIMP-1*), two well-known factors involved in EMT^26–28^. The third most upregulated gene was *TBL1* (Fig. 1a). TBL1 together with its paralogous partner TBLR1 regulate cofactor exchange at nuclear receptor genes^29^. TBL1 and TBLR1 also control β-catenin-mediated regulation of Wnt target genes^25^; however, the role of TBL1 in regulation of epithelial genes and EMT has not been previously investigated. *TBL1* mRNA levels increased 46-fold in HMEC-RAS-ZEB1 versus HMEC-RAS by reverse transcription–quantitative real-time polymerase chain reaction (RT-qPCR) (Fig. 1b), confirming the microarray data. Therefore, we selected this protein for a deep characterization of its role in the mesenchymal phenotypes. First, we determined TBL1 protein expression levels in HMEC-RAS-ZEB1 and HMEC-RAS cells by western blotting and immunofluorescence. TBL1 protein levels were strongly increased (30-fold increase) in the cell line overexpressing mZEB1 with respect to the control cell line (Fig. 1c, d). In contrast, the levels of TBLR1 were not significantly changed. Levels of ZEB1, CDH1 (epithelial marker), and VIM (mesenchymal marker) were also determined as controls. Figure 1b shows that, in addition to the *HA-mZEB1 mRNA*, HMEC-RAS-ZEB1 cells also presented increased levels of the endogenous *hZEB1* mRNA. Western blotting and immunofluorescence using an anti-ZEB1 that recognizes both HA-ZEB1 and hZEB1 demonstrated increased levels of ZEB1 protein in HMEC-RAS-ZEB1 respect to HMEC-RAS cells, as expected (Fig. 1c, d). Levels of CDH1 protein were much lower in HMEC-RAS-ZEB1 than in HMEC-RAS. Inversely, VIM levels increased in HMEC-RAS-ZEB1 cells with respect to HMEC-RAS cells. Interestingly, TBL1 was also upregulated 48 h upon induction of EMT by TGF-β treatment in mouse mammary epithelial cells NMuMG (Fig. 1e), suggesting that TBL1 upregulation is a common feature of the mesenchymal state.

### TBL1 represses *CDH1* expression

**Fig. 2:**
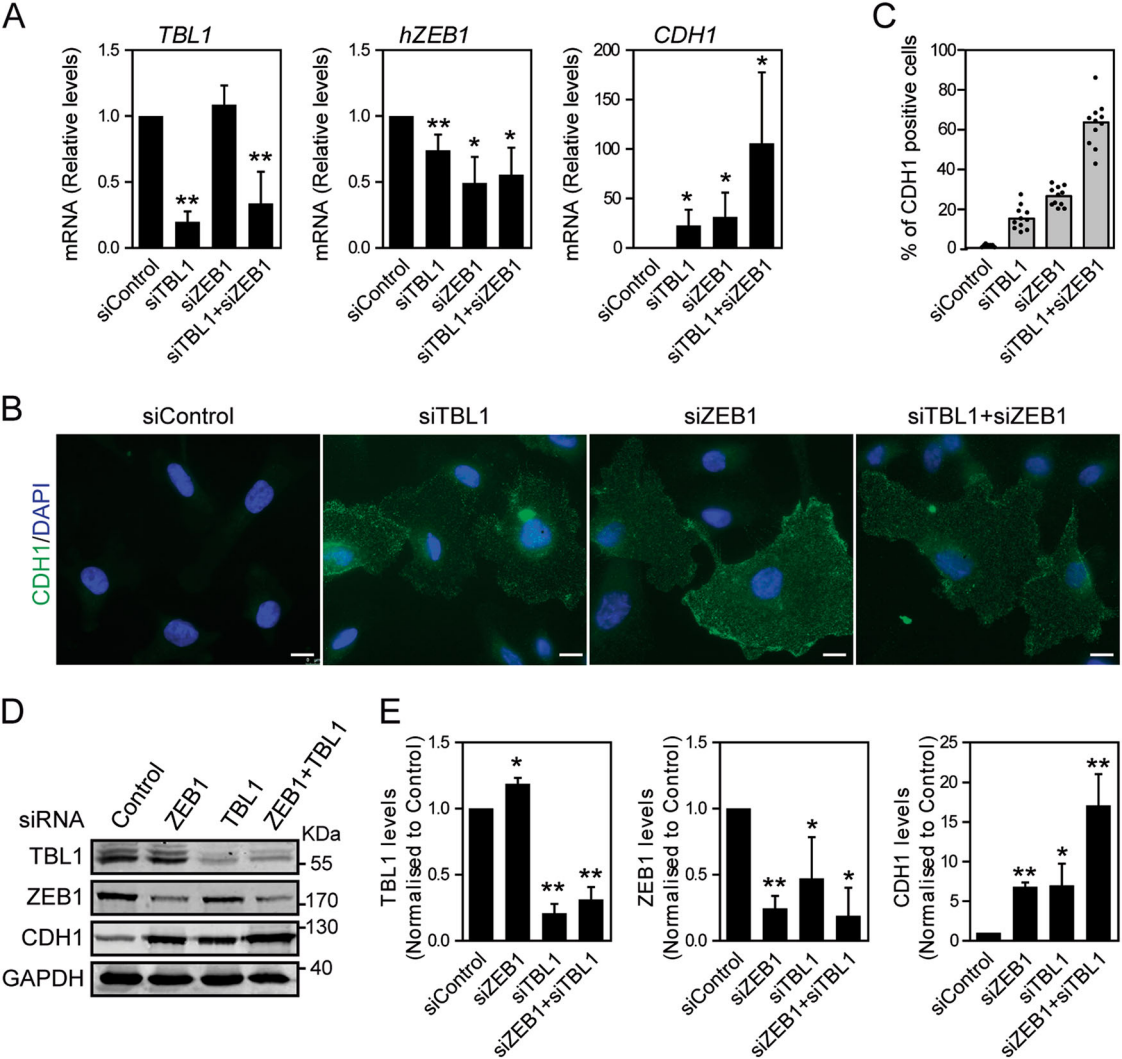
TBL1 cooperates with ZEB1 to repress *CDH1* gene expression. **a** Depletion of TBL1 and ZEB1 synergistically increases *CDH1* gene expression. *TBL1, hZEB1*, and *CDH1* mRNA expression was analyzed by reverse transcription–quantitative real-time polymerase chain reaction in HMEC-RAS-ZEB1 cells 72 h after transfection with the indicated small interfering RNAs (siRNAs). Data are the average of *n* = 6 data from three independent experiments ± SD. *P < 0.05; **P < 0.01 versus control by analysis of variance (ANOVA). **b**–**e** Depletion of TBL1 increases CDH1 protein expression. **b** Immunofluorescence staining of CDH1 in HMEC-RAS-ZEB1 cells transfected with the indicated siRNAs. Nuclei were stained with 4,6-diamidino-2-phenylindole. Bar, 10 μm. **c** Quantification of immunofluorescence experiments shown in **b**. Total number of scored cells per condition ≥200. **d** HMEC-RAS-ZEB1 cells were transfected with the indicated siRNAs. At 72 h after transfection, a total lysate was prepared and analyzed by western blot for the indicated proteins. **e** Quantification of TBL1, ZEB1, and CDH1 levels, normalized with respect to GAPDH expression. Data are the average of three independent experiments ± SD. *P < 0.05; **P < 0.01 versus control by ANOVA

The E-cadherin encoding gene (*CDH1*) is a key epithelial marker, and repression of this gene is a hallmark of the EMT process^13^. Therefore, we tested whether TBL1 is involved in controlling the expression of *CDH1*. Knockdown of TBL1 in HMEC-RAS-ZEB1 cells by interference RNA promoted upregulation of CDH1 at both the mRNA and protein levels (Fig. 2). The *CDH1* transcript was also upregulated with a second independent small interfering RNA (siRNA) that targeted *TBL1* (Supplementary Fig. S1A). Knockdown of ZEB1 by a siRNA that targets both the endogenous *hZEB1* (Fig. 2a and Supplementary Fig. S1B) and the transgenic *HA-mZEB1* (Supplementary Fig. S1B) was also performed as control. Depletion of ZEB1 also promoted the upregulation of *CDH1*, as expected (Fig. 2a–e). Notably, knockdown of TBL1 or ZEB1 increased the *CDH1* transcript levels by 22.9- and 31.3-fold, respectively, while knockdown of both TBL1 and ZEB1 at the same time increased transcript levels by 105.8-fold, indicating that both factors synergistically cooperate to regulate *CDH1* (Fig. 2a). Synergistic upregulation was also observed at the level of protein levels (6.8- and 7.0-fold, respectively, after independent knockdown, versus 17.1-fold after combined knockdown) (Fig. 2c, d). Depletion of TBL1 also decreased endogenous hZEB1 expression at both the mRNA and protein levels, suggesting that TBL1 directly or indirectly also controls hZEB1 levels.

### TBL1 cooperates with ZEB1 to regulate the *CDH1* and *hZEB1* promoters

**Fig. 3:**
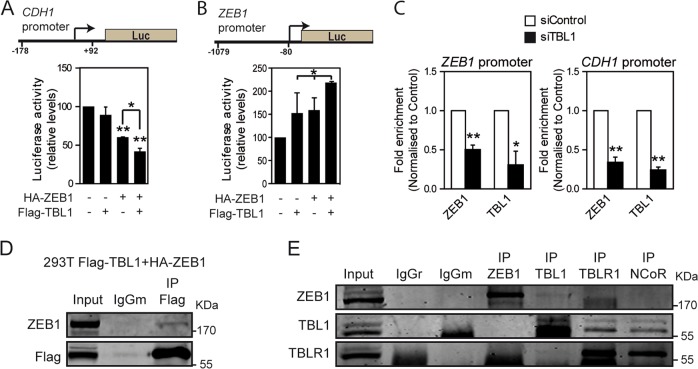
TBL1 and ZEB1 interact and cooperate for regulation of the *CDH1* and *ZEB1* promoters. **a** Reporter assay showing repression of the *CDH1* promoter by Flag-TBL1 and HA-ZEB1 in NMuMG cells. **b** Reporter assay showing activation of the *ZEB1* promoter by Flag-TBL1 and HA-ZEB1 in HEK293T cells. **c** TBL1 and ZEB1 bind to the *CDH1* and *ZEB1* promoters. HMEC-RAS-ZEB1 cells transfected with control siRNAs (siControl) or siRNAs against TBL1 (siTBL1) were subjected to chromatin immunoprecipitation assays with the indicated antibodies. Fold enrichment indicate occupancies relative to siControl. **a**–**c** Values are the average of *n* = 6 data from three independent experiments ± SD. *P < 0.05; **P < 0.01, as compared to control or between the indicated samples, determined by analysis of variance. **d**, **e** TBL1 interacts with ZEB1 and CDH1. **d** HEK293T cells were transfected with expression vectors encoding Flag-TBL1 and HA-ZEB1. Cell extracts were subjected to immunoprecipitation with anti-Flag or control (IgG) antibodies and analyzed by Western blot for the presence of the indicated proteins. **e** Whole cell extracts from HMEC-RAS-ZEB1 cells were subjected to immunoprecipitation with anti-ZEB1, anti-TBL1, anti-TBLR1, anti-NCoR, or control (IgG) antibodies. Immunoprecipitated proteins and 3% of the input were analyzed by western blot with the indicated antibodies

Next, we investigated whether TBL1 directly regulates the *CDH1* promoter. We used a reporter plasmid in which expression of the luciferase gene is under the control of the *CDH1* promoter (region –178 to +92 with respect to the transcriptional start site [TSS]). As expected, co-transfection of an HA-ZEB1 expression plasmid with the *CDH1* promoter reporter construct reduced luciferase activity levels (Fig. 3a). Interestingly, in the absence of ZEB1, Flag-TBL1 did not repress the *CDH1* promoter; however, co-expression of Flag-TBL1 increased repression caused by HA-ZEB1, suggesting that TBL1 cooperates with ZEB1 to repress the *CDH1* promoter (Fig. 3a). We have also shown that knockdown of TBL1 decreases the expression of endogenous hZEB1 (Fig. 2). Therefore, we tested whether TBL1 regulates the *ZEB1* promoter (region −1079 to −80 with respect to the TSS), also by using a luciferase reporter plasmid. In this case, expression of Flag-TBL1 or HA-ZEB1 increased luciferase activity (Fig. 3b). Co-expression of both factors together further increased activity, again suggesting a cooperation between both factors (Fig. 3b).

We then performed chromatin immunoprecipitation (ChIP) experiments using HMEC-RAS-ZEB1 cells to investigate whether endogenous TBL1 and ZEB1 are directly recruited to the *CDH1* and *ZEB1* promoters. We found that both TBL1 and ZEB1 were present at both promoters (Fig. 3c). As a control, we verified that TBL1 depletion decreased TBL1 occupancy, confirming the specificity of the ChIP signals. Interestingly, TBL1 knockdown also decreased the occupancy of ZEB1 at both the *CDH1* and *ZEB1* promoters (Fig. 3c), consistent with the reduction of ZEB1 levels observed upon TBL1 depletion (Fig. 2).

We next tested whether TBL1 interacts physically with ZEB1 by co-immunoprecipitation of transiently co-expressed Flag-tagged TBL1 and HA-tagged ZEB1. Indeed, HA-ZEB1 co-immunoprecipitated with Flag-TBL1 (using anti-flag antibodies) (Fig. 3d). Furthermore, endogenous ZEB1 also co-immunoprecipitated with endogenous TBL1 protein in HMEC-RAS-ZEB1 cells (Fig. 3e). As previously described, we found that TBL1 co-immunoprecipitated with NCoR1 and TBLR1. However, we did not find that ZEB1 co-immunoprecipitated with either NCoR1 or TBLR1. This fact may suggest that the TBL1–ZEB1 interaction is partially independent of the NCoR complex or that ZEB1 interacts with the complex through TBL1. In summary, our data suggest that the cooperation between ZEB1 and TBL1 for controlling the *CDH1* and *ZEB1* promoters occurs through a physical interaction.

### TBL1 is essential for the mesenchymal phenotype of HMEC-RAS-ZEB1 cells

Our results suggest that TBL1 is important for repression of the *CDH1* gene and for activation of *ZEB1* gene, two important players of the mesenchymal phenotype. In agreement, HMEC-RAS-ZEB1 cells depleted of TBL1 displayed a decreased mesenchymal-like morphology that resemble the HMEC-RAS cellular morphology (Supplementary Fig. S2a). Depletion of TBL1 in HMEC-RAS-ZEB1 cells caused the expression of *CXADR* and *CLDN12* genes, two well-known epithelial markers (Supplementary Fig. S2b). In addition, depletion of TBL1 provoked a small reduction of cell proliferation (Supplementary Figure S3a) that could not be correlated with changes in cell cycle (Supplementary Figure S3b and S3c) and a small but significant increase of apoptosis from 2.5 to 5.5% of the cells (Supplementary Figure S3d). Depletion of ZEB1 provoked similar effects on cell proliferation and apoptosis.

**Fig. 4:**
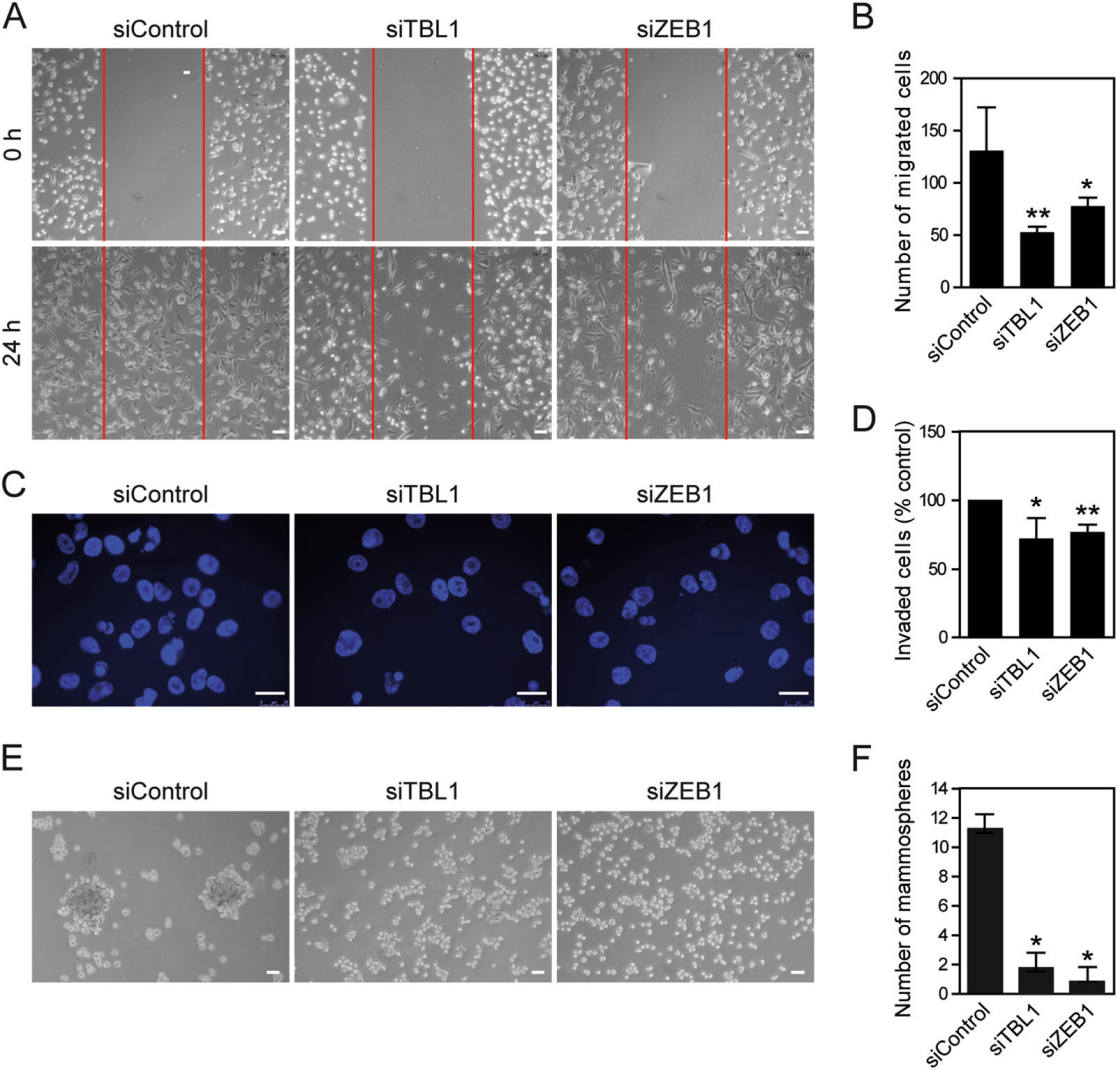
TBL1 is required for migration, invasion, and mammosphere-formation phenotypes of HMEC-RAS-ZEB1 cells. **a**, **b** TBL1 depletion inhibits cell migration in scratch assays. **a** HMEC-RAS-ZEB1 cells were transfected with the indicated siRNAs. Cells were plated 72 h after transfection onto culture inserts, which were removed 24 h later. Phase-contrast pictures of the wounds at different locations were taken at the time of removal and again 24 h later. **b** Quantification of the representative scratch assays shown in **a**. Cells in the wound area were counted over four microscopic fields for each condition. **c**, **d** TBL1 is required for HMEC-RAS-ZEB1 invasiveness. HMEC-RAS-ZEB1 cells were transfected with the indicated siRNAs. At 72 h after transfection, identical number of cells of each treatment were deposited on Matrigel-coated filters and allowed to invade during 18 h. The migrating cells were then stained and visualized by microscopy. Duplicate filters were counted in three individual experiments (**d**). **e** Mammosphere-formation assay under low adherence conditions. **f** Quantification of mammosphere-formation assay. **b**, **d**, **f** Values are average of three independent experiments ± SD. *P < 0.05, **P < 0.01 as compared to control and determined by analysis of variance

Next, we checked whether TBL1 knockdown affects cell motility and invasiveness of HMEC-RAS-ZEB1 cells, two characteristics of mesenchymal cells. We also monitored the effect of ZEB1 knockdown as a control. Scratch assays demonstrated that TBL1 depletion decreased cell motility to a similar extent as ZEB1 depletion (Fig. 4a, b). Cell invasion was monitored by using Matrigel-coated Boyden chambers. Both TBL1 and ZEB1 depletion impaired invasiveness also to a similar extent in this assay (Fig. 4c, d).

Upon EMT, certain cell types acquire stem cell-like properties^5,6^. In fact, HMEC-RAS-ZEB1 cells are able to form mammospheres^22^, a property associated with mammary epithelial stem cells^30^. Interestingly, TBL1 depletion decreased the ability of HMEC-RAS-ZEB1 cells to form mammospheres (Fig. 4e, f). As expected, ZEB1 depletion also inhibited the mammosphere-formation ability. Taken together, these data indicate that high levels of TBL1 are required for the maintenance of mesenchymal and at least one of the stem cell-like properties of HMEC-RAS-ZEB1 cells.

### *TBL1* in breast cancer

**Fig. 5:**
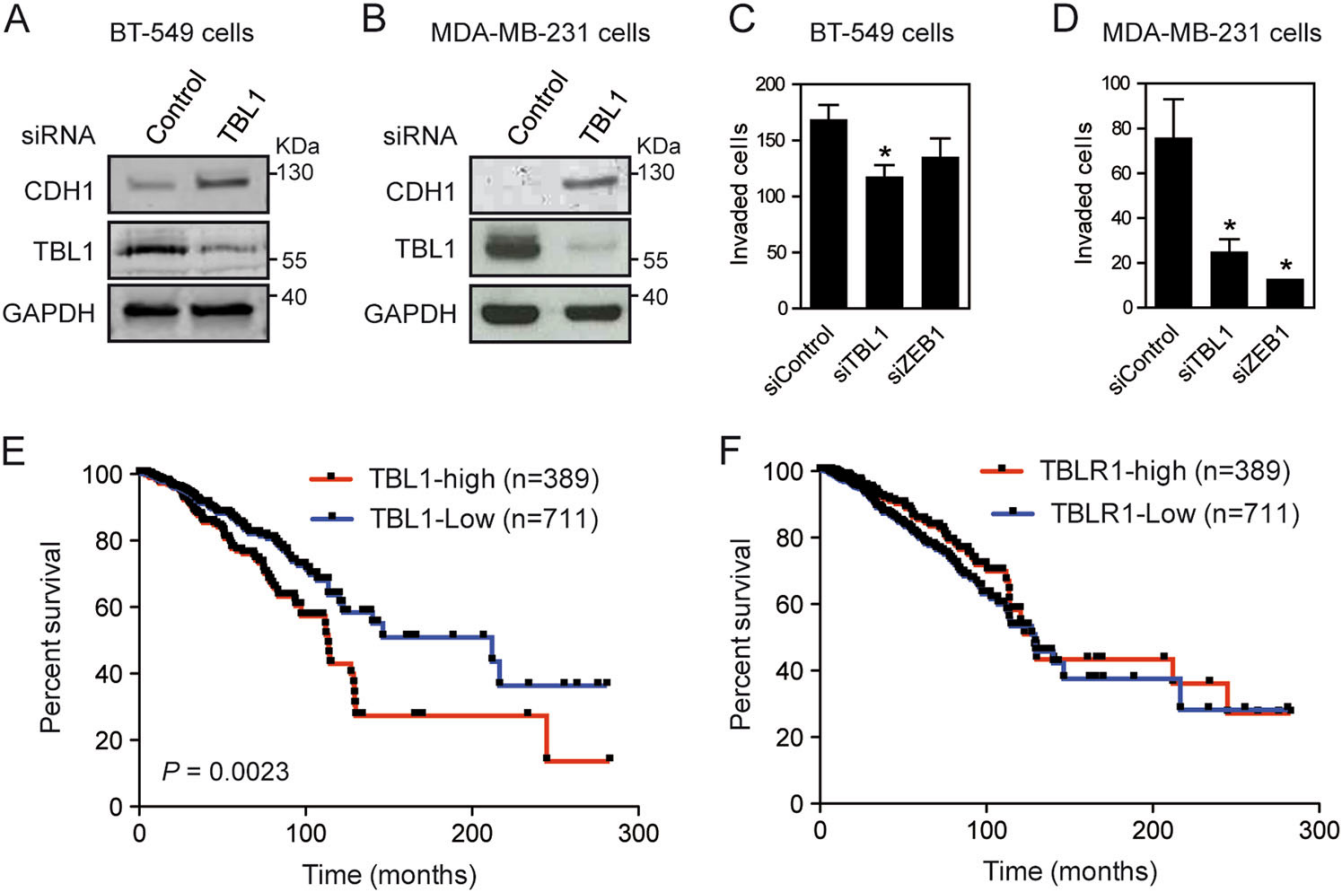
TBL1 is required for mesenchymal traits in breast cancer cells. **a**, **b** Depletion of TBL1 increases levels of CDH1 protein in BT-549 (**a**) and MDA-MB-231 cells (**b**). Cells were transfected with the indicated small interfering RNAs (siRNAs). At 72 h after transfection, CDH1, TBL1, and GAPDH expression was analyzed by western blot. **c**, **d** TBL1 depletion inhibits cell invasion in BT-549 (**c**) and MDA-MB-231 cells (**d**). Cells were transfected with the indicated siRNAs, and cell motility through Matrigel-coated filters was measured at 18 h after plating. The migrating cells were then stained and visualized by microscopy. Duplicate filters were counted in three individual experiments. Depletion of ZEB1 was performed as a control. Values are the average of three independent experiments ± SD. *P < 0.05 as compared to control and determined by analysis of variance. **e**, **f** Kaplan–Meier survival plots for breast carcinoma patients from The Cancer Genome Atlas. Patients were divided into subgroups according to the *TBL1* (**e**) or *TBLR1* (**f**) mRNA expression (RNA-seq data). *P* values were calculated by log-rank test

HMEC-RAS-ZEB1 cells exhibit characteristics of claudin-low breast tumor cells^22^. We thus investigated the role of TBL1 in two breast cancer cell lines with claudin-low characteristics: MDA-MB-231 and BT-549^31^. In agreement with the data obtained with HMEC-RAS-ZEB1, downregulation of TBL1 in both cell lines significantly increased the levels of CDH1 protein (Fig. 5a, b). Furthermore, depletion of TBL1 also decreased cell invasiveness of both cell lines. Similar results have been reported in the cell line MDA-MB-231 using a lentiviral-based silencing strategy^32^. These data confirm that TBL1 is required for the mesenchymal traits of cell lines with claudin-low characteristics (Fig. 5c, d).

These results made us to explore whether TBL1 might have a role in breast cancer. For that, RNA-seq and clinicopathological data of the breast invasive carcinoma cohort from The Cancer Genome Atlas (TCGA) were analyzed. Levels of *TBL1* transcript expression were not significantly higher in normal tissue than in cancer tissue samples. We next divided the tumor samples into two groups according to the *TBL1* gene expression and compared survival plots of patients with TBL1-low tumors with those of patients with TBL1-high tumors. Strikingly, patients with TBL1-high tumors presented a median survival of 113.7 months, while patients with TBL1-low tumors presented a median survival of 212.1 months (log-rank test *P* value = 0.0023), indicating that a high expression of *TBL1* transcript is associated with poor prognosis (Fig. 5e). No differences in prognosis were associated with high versus low levels of *TBLR1* transcript (Fig. 5f). We then investigated whether expression levels of the *TBL1* gene was associated with specific clinicopathological factors such as sex, age, tumor stage (T1 to T4 and stage I to IV), lymph node metastasis (N), or distant metastasis (M) (Table 1). Interestingly, we observed a significantly higher proportion of stage IV tumors in the group of patients with TBL1-high tumors (*P* = 0.033). Likewise, distant metastasis (M1) was also more abundant in the TBL1-high than in the TBL1-low patient group (*P* = 0.041). No significant differences in clinicopathological factors were associated with high versus low levels of TBLR1.

**Table 1:** Association between *TBL1* and *TBLR1* mRNA expression and clinicopathological factors in the breast carcinoma dataset from TCGA

Factor	*TBL1* expression			*TBLR1* expression		
	High expression, *n* = 389	Low expression, *n* = 710	*P* value	High expression, *n* = 389	Low expression, *n* = 710	*P* value
Age	58.67 ± 14.1	58.19 ± 12.7	0.53^a^	57.50 ± 12.9	58.8 ± 13.4	0.11^a^
Sex			1^b^			0.67^b^
Male	389 (98.9%)	697 (98.9%)		389 (98.7%)	698 (99.0%)	
Female	4 (1.0%)	8 (1.1%)		5 (1.3%)	7 (1.0%)	
Tumor stage (T)			0.305^b^			0.061^b^
T1+T2	319 (82.2%)	600 (84.6%)		341 (86.6%)	578 (82.2%)	
T3+T4	69 (17.8%)	109 (15.4%)		53 (13.5%)	125 (17.8%)	
Tumor stage (S)			**0.033** ^b^			0.13^b^
SI+SII+SIII	367 (96.9%)	689 (98.7%)		384 (99.0%)	676 (97.7%)	
SIV	12 (3.1%)	9 (1.3%)		4 (1.0%)	16 (2.3%)	
Lymph node metastasis			0.2^b^			0.069^b^
N0	172 (45.0%)	344 (49.0%)		200 (51.4%)	315 (45.7%)	
N1+N2+N3	210 (55.0%)	358 (51.0%)		189 (48.6%)	375 (54.3%)	
Metastasis stage code			**0.041** ^b^			0.13^b^
M0	325 (96.4%)	590 (98.5 %)		354 (98.6%)	561 (97.1%)	
M1	12 (3.6%)	9 (1.5%)		5 (1.4%)	17 (2.9%)	

^a^Student's *t* test

^b^Chi-square test. Significant *P* values (*P* ≤ 0.05) are depicted in bold

## Discussion

In this manuscript, we analyze a number of chromatin and epigenetic factors that are upregulated in a mesenchymal transformed cell lines with respect to its parental epithelial counterpart. Among the upregulated factors, we found components of the SWI/SNF complex (SMARCA4, SMARCA2, ARID1A) and the Polycomb complex (EED, SUZ12 and BMI), two complexes well known to be involved in the EMT process^18,21^. Other upregulated factors, such as LOX and PRDM1, have been previously associated to EMT^26–28^. One of the factors that changed drastically its expression levels was TBL1, a well-known coregulator of nuclear receptors and β-catenin^23,25^, whose involvement in EMT is not characterized. We have investigated the involvement of TBL1 in *CDH1* repression and maintenance of the mesenchymal state. We show that TBL1 regulates the mesenchymal phenotype both by acting as a corepressor of the *CDH1* gene with ZEB1 and by acting positively on ZEB1 autoactivation. In addition, ChIP experiments demonstrated that TBL1 binds to both *CDH1* and *ZEB1* endogenous promoters. Interestingly, depletion of TBL1 strongly decreased ZEB1 occupancy at the *CDH1* and *ZEB1* promoters, which is consistent with the reduction of ZEB1 levels we observed after TBL1 knockdown. It is also possible that TBL1 has a role in stabilizing ZEB1 at the chromatin of both gene promoters.

How does TBL1 behave at the same time as a coactivator and a corepressor? It has been shown that ZEB1 can act as a repressor by interacting with and recruiting the chromatin factors CtBP and BRG1, but it also can act as an activator through its interaction with the acetyltransferase P300^20,21,33,34^. Interestingly, our data suggest that, in contrast to the previously identified chromatin coregulators, TBL1 cooperates with ZEB1 for both its activation and repression functions. It is unclear how ZEB1 turns from a repressor to an activator; however, recent studies suggest that its conversion to an activator requires the action of the Wnt/β-catenin pathway^35^. TBL1 was initially described as a subunit of the repressor complex NCoR^23^. It soon became clear, however, that TBL1 and TBLR1 mediate exchange of the nuclear receptor corepressors NCoR and SMRT for coactivators upon ligand binding^29^; they were thus termed exchange factors. TBL1 recruits β-catenin to Wnt target genes for activation^36^, and ZEB1 is activated by the Wnt/β-catenin pathway^37^. We now show here that TBL1 interacts directly with ZEB1. Therefore, it is possible that TBL1 mediates ZEB1-dependent repression of *CDH1* through the NCoR complex and that it also mediates activation of *ZEB1* gene expression through its ability to interacts with β-catenin. This exemplifies a novel exchange activity of TBL1 that, in this case, is important for the stabilization of the mesenchymal phenotype of HMEC-RAS-ZEB1 cells. Consequently, TBL1 depletion impairs motility, invasiveness, and mammosphere-formation phenotypes of HMEC-RAS-ZEB1 cells.

Interestingly, TBL1 depletion also reduces mesenchymal traits of breast cancer cell lines of the claudin-low subtype. Claudin-low breast tumors are associated with early metastasis and resistance to chemotherapy, and its transcriptome is characterized by the expression of markers of EMT^31,38–40^. Consistent with an important role of TBL1 in regulating EMT, and with a role of EMT in tumor dissemination in at least certain types of breast cancer, we observed that patients who had tumors with high levels of TBL1 also presented a higher proportion of metastasis. This fact is in agreement with a poor prognosis of patients presenting breast tumors with high levels of TBL1. High levels of TBL1 have also been recently associated with poor prognosis in pancreatic ductal adenocarcinoma^24^, suggesting that TBL1 is an important prognosis marker in several cancer types.

In summary, our data contribute to emphasizing the critical involvement of different chromatin regulatory factors in the transcriptional reorganization that occurs during EMT. We have characterized here the important role of TBL1 in maintaining the mesenchymal phenotype in cell lines with claudin-low characteristics. How the different factors are coordinated among themselves, and why a high number of chromatin regulatory machineries are required to control one single gene such as *CDH1*, are interesting questions to be addressed in future.

## Materials and methods

### Cells and treatments

Human mammary epithelial cells HMEC-RAS and HMEC-RAS-ZEB1, kindly provided by Alain Puisieux (Lyon, France) in 2014, were grown in Dulbecco’s modified Eagle’s medium (DMEM)/F12 complemented with 10% fetal bovine serum (FBS), 10 ng/mL epidermal growth factor (EGF), 0.5 µg/mL hydrocortisone, and 10 µg/mL insulin. The human breast cancer cell line MDA-MB-231 (provided by Abelardo Lopez-Rivas, CABIMER, in 2014) and human embryonic kidney cell line 293T (provided by Rosa Ríos, CABIMER, in 2010) were grown in DMEM supplemented with 10% FBS. Normal murine mammary gland NMuMG cells (provided by José Antonio Pintor-Toro, CABIMER, in 2014) were cultured in DMEM medium supplemented with 10% FBS and 10 µg/mL insulin. BT-549 cells (provided by Abelardo Lopez-Rivas, CABIMER, in 2017) were cultured in DMEM/F12 medium supplemented with 10% FBS and 10 µg/mL insulin. All cell lines were tested for mycoplasma infection once per year using the MycoAlert Detection Kit (Lonza). Before freezing, the stocks cells were treated with the anti-*mycoplasma* antibiotics Plasmocin (InvivoGen). All cell lines were propagated no more than 15 passages after defrosting the stocks and not further authenticated.

Transient EMT was induced by the addition of TGF-β1 (10 ng/mL; R&D Systems) for 48 h.

### Microarray data and selection of chromatin and epigenetic factors

Relative mRNA levels of 824 genes encoding epigenetic and chromatin factors of HMEC-RAS-ZEB1 and HMEC-RAS cell lines were obtained from GEO accession GSE32727^22^ (Supplementary Table S1). The selection of 824 chromatin and epigenetic factors was carried out by combining epigenetic factors of the EpiFactors database^41^ and a previously published list of factors^42^ containing the following protein domains or functions: PHD, BROMO, CHROMO, PWWP, tandem BRCT, TUDOR, BAH, MBT, SET, JMJC, JMJN, PRMT, HAT, HDAC, SIRT, DNMT, MBD, and SNF2 ATP-dependent remodelers.

### DNA constructs

The full-length human Flag-TBL1 expression plasmid was kindly provided by Cun-Yu Wang^25^; the full-length human HA-ZEB1 expression plasmid by Thomas Brabletz^43^; the *CDH1* gene promoter reporter plasmid pGL3-E-cad (−178 to +92) by A. García de Herreros^44^; and the *ZEB1* gene promoter (–1079 to –80) reporter plasmid pGL4.15-ZEB1 by Min Yu^45^.

### Transfections and luciferase reporter assays

Transient transfections of cells with expression or reporter plasmids were performed with Lipofectamine (Invitrogen) or FuGENE HD (Promega) for 293T cells. For siRNA transfections, we use Oligofectamine (Invitrogen), for HMEC-ZEB1 cells, and RNAiMAX (Invitrogen), for NMuMG cells. The siRNA sequences are shown in Supplementary Table S2. siRNA against ZEB1 was designed to target both the *hZEB1* and the *HA-mZEB1* mRNAs.

Luciferase assay were performed as previously described^19^. The total amount of DNA in each individual well was kept constant by adding empty vector (Flag or HA) as appropriated.

### Antibodies, western blotting, immunoprecipitation, and immunohistochemistry

Co-immunoprecipitation, immunohistochemistry, and western blotting were performed as previously described^46^. The antibodies used for immunoprecipitation and western blot were: mouse monoclonal anti-Flag (M2, F1804, Sigma-Aldrich), rabbit polyclonal anti-ZEB1 (H-102, Santa Cruz), mouse monoclonal anti-TBL1 (F-2, Santa Cruz), rabbit polyclonal anti-TBLR1 (Bethyl), mouse monoclonal anti-VIM (v5255, Sigma), mouse monoclonal anti-E-cadherin (BD Transduction Laboratories, BD Biosciences, San Jose, CA, USA), mouse monoclonal anti-GAPDH (6C5, Santa Cruz), and mouse monoclonal anti-α-tubulin (T9026, Sigma-Aldrich). The secondary antibodies used were IRDye 680 goat anti-mouse IgG (H+L) antibody (926-68070, LiCOR) and IRDye 800 goat anti-rabbit IgG (H+L) antibody (926-32211, LiCOR). Antibodies used in ChIP experiments were the rabbit polyclonal anti-ZEB1 (H-102, Santa Cruz) and the mouse monoclonal anti-TBL1 (Ab24548, Abcam). Antibodies used in immunofluorescence were the mouse monoclonal anti-TBL1 (F-2, Santa Cruz), rabbit polyclonal anti-ZEB1 (H-102, Santa Cruz), and the mouse monoclonal anti-E-cadherin (67A4, Santa Cruz). Secondary antibodies used were Texas Red dye-conjugated goat anti-mouse IgG (Jackson ImmunoResearch) and fluorescein isothiocyanate-conjugated goat anti-mouse IgG (Molecular Probes). For quantification of immunofluorescences shown in Fig. 2b, 11 different microscopic fields per condition were counted and the percentage of CDH1-expressing cells with respect to the total number of cells were represented. Total number of scored cells per condition ≥200.

### Growth curves, cell cycle, and apoptosis analysis

For cell growth analysis, 10^5^ cells were seeded in 6-well plates. Every 24 h, cells of a well were collected and counted using a Neubauer hemocytometer up to *t* = 5 days. At *t* = 0, 3, and 5 days, silencing was monitored by RT-qPCR. Cell cycle and apoptosis were determined by flow cytometry. For that, cells were harvested, washed with phosphate-buffered saline (PBS) and resuspended in ice-cold PBS. Ethanol (70%) was added dropwise while vortexing at low speed, and cells were then fixed at 4 °C for at least 1 h. Cells were washed with PBS and treated with fluorescence-activated cell sorting (FACS) buffer (250 μg/mL RNase A [Sigma-Aldrich], and 10 μg/mL propidium iodide diluted in PBS). Cells were incubated at 37 °C for 30 min and then analyzed using a FACSCalibur (BD). Apoptosis was determined by measuring the percentage of cells containing a subG1 DNA content.

### Scratch assays and invasion assays

Scratch assays were performed as previously described^19^. At 24 h after transfection with either control, ZEB1, or TBL1 siRNAs, an equal number of cells were plated into culture inserts (Ibidi, Martinsried, Germany) and incubated at 37 °C for 24 h. The next day, the culture inserts were removed, and the wound was imaged at 0 and 24 h. To monitor cell invasion, Boyden chambers assays were used as previously described^47^, with 24-well Transwell 8-µm filters that were coated with 10 µg growth factor-reduced Matrigel (Trevigen). Complete medium was used as a chemoattractant. At 72 h after transfection with either siControl, siZEB1, or siTBL1, cells were trypsinized and counted. Then cell suspension containing 10^5^ cells in 0.5 ml Dulbecco's Modified Eagle's Medium and 0.1% bovine serum albumin were placed into the upper compartment of the Boyden chamber. Cell were allowed to invade for 18 h. Then cells on the filter top surface were removed and cells that had penetrated the filters were nuclear-stained with 4,6-diamidino-2-phenylindole. After the nuclei were counted, invaded cells were expressed as the average number of migrated cells bound per microscopic field over seven fields per assay in triplicate experiments.

### Mammosphere formation

For mammosphere formation, single cells were plated on a 60-mm ultra-low attachment tissue culture dish (Corning, Lowell, MA, USA) at a density of 10^5^ cells/mL in DMEM/F12 complemented with 10% FBS, 10 ng/mL EGF, 0.5 hydrocortisone, and 10 µg/mL insulin and incubated for 8 days.

### RNA extraction and RT-qPCR

Total RNA was prepared by using the RNeasy Kit (Qiagen), as described in the manufacturer’s instructions, including DNase I digestion to avoid potential DNA contamination. cDNA was generated from 100 ng of total RNA by using SuperScript First Strand Synthesis System (Invitrogen). Two µg of generated cDNA solution was used as a template for real-time PCR (qPCR). Gene products were quantified by qPCR with the Applied Biosystems 7500 FAST Real-Time PCR System, using Applied Biosystems Power SYBR Green Master Mix. Values were normalized to the expression of the *GAPDH* housekeeping gene. Each experiment was performed at least in triplicate. All oligonucleotide sequences used are listed in Supplementary Table S3.

### ChIP assays

ChIP assays were performed as previously described^48^. Quantification of immunoprecipitated DNA was performed by qPCR. ChIP was quantified by using three qPCR determinations. Provided data are the average of at least three independent experiments. All oligonucleotide sequences used are listed in Supplementary Table S3.

### Analysis of TCGA data

RNA-seq expression data and clinical data of the breast carcinoma cohort were obtained from The Cancer Genome Atlas (http://cancergenome.nih.gov/)^49^. Survival plots and the log-rank test were performed using GraphPad *Prism* version 5.0. For tumor description, the Tumor–Node–Metastasis (TNM) staging system (www.cancerstaging.org/) was used, whereby T followed by a number (1–4) describes the size of the tumor (with T4 being the largest); N followed by 1 or 0 indicates whether lymph nodes have metastasis or not, respectively; and M followed by 1 or 0 indicates whether the tumor has metastasized or not, respectively. We also considered the roman numeral stage annotation (S) from I to IV, with each number corresponding approximately to a combination of the TNM numbers. No subdivisions of stages were used. Significance of differences between groups were computed using Prism 5 (GraphPad) and either Student’s *t* test or Chi-square test, with confidence interval of 95%.

## Supplementary information

Supplementary Fig S1

Supplementary Fig S2

Supplementary Fig S3

Supplementary Table S1

Supplementary Table S2

Supplementary Table S3

## References

[1] Nieto, M. A., Huang, R. Y., Jackson, R. A. & Thiery, J. P. EMT: 2016. *Cell* **166**, 21–45 (2016). 10.1016/j.cell.2016.06.028 27368099

[2] Nieto MA (2013). Epithelial plasticity: a common theme in embryonic and cancer cells. Science.

[3] Beerling E (2016). Plasticity between epithelial and mesenchymal states unlinks EMT from metastasis-enhancing stem cell capacity. Cell Rep..

[4] Yu M (2013). Circulating breast tumor cells exhibit dynamic changes in epithelial and mesenchymal composition. Science.

[5] Morel AP (2008). Generation of breast cancer stem cells through epithelial-mesenchymal transition. PLoS ONE.

[6] Mani SA (2008). The epithelial-mesenchymal transition generates cells with properties of stem cells. Cell.

[7] Fischer KR (2015). Epithelial-to-mesenchymal transition is not required for lung metastasis but contributes to chemoresistance. Nature.

[8] Zheng X (2015). Epithelial-to-mesenchymal transition is dispensable for metastasis but induces chemoresistance in pancreatic cancer. Nature.

[9] Krebs AM (2017). The EMT-activator Zeb1 is a key factor for cell plasticity and promotes metastasis in pancreatic cancer. Nat. Cell Biol..

[10] Ye X, Weinberg RA (2015). Epithelial-mesenchymal plasticity: a central regulator of cancer progression. Trends Cell Biol..

[11] Ye X (2017). Upholding a role for EMT in breast cancer metastasis. Nature.

[12] Lambert AW, Pattabiraman DR, Weinberg RA (2017). Emerging biological principles of metastasis. Cell.

[13] Lamouille S, Xu J, Derynck R (2014). Molecular mechanisms of epithelial-mesenchymal transition. Nat. Rev. Mol. Cell Biol..

[14] Wu CY, Tsai YP, Wu MZ, Teng SC, Wu KJ (2012). Epigenetic reprogramming and post-transcriptional regulation during the epithelial-mesenchymal transition. Trends Genet..

[15] Tam WL, Weinberg RA (2013). The epigenetics of epithelial-mesenchymal plasticity in cancer. Nat. Med..

[16] Lin Y (2010). The SNAG domain of Snail1 functions as a molecular hook for recruiting lysine-specific demethylase 1. EMBO J..

[17] Lin T, Ponn A, Hu X, Law BK, Lu J (2010). Requirement of the histone demethylase LSD1 in Snai1-mediated transcriptional repression during epithelial-mesenchymal transition. Oncogene.

[18] Herranz N (2008). Polycomb complex 2 is required for E-cadherin repression by the Snail1 transcription factor. Mol. Cell Biol..

[19] Rivero S, Ceballos-Chavez M, Bhattacharya SS, Reyes JC (2015). HMG20A is required for SNAI1-mediated epithelial to mesenchymal transition. Oncogene.

[20] Postigo AA, Dean DC (1999). ZEB represses transcription through interaction with the corepressor CtBP. Proc. Natl. Acad. Sci. USA.

[21] Sanchez-Tillo E (2010). ZEB1 represses E-cadherin and induces an EMT by recruiting the SWI/SNF chromatin-remodeling protein BRG1. Oncogene.

[22] Morel AP (2012). EMT inducers catalyze malignant transformation of mammary epithelial cells and drive tumorigenesis towards claudin-low tumors in transgenic mice. PLoS Genet..

[23] Guenther MG (2000). A core SMRT corepressor complex containing HDAC3 and TBL1, a WD40-repeat protein linked to deafness. Genes Dev..

[24] Stoy C (2015). Transcriptional co-factor Transducin beta-like (TBL) 1 acts as a checkpoint in pancreatic cancer malignancy. EMBO Mol. Med.

[25] Li J, Wang CY (2008). TBL1-TBLR1 and beta-catenin recruit each other to Wnt target-gene promoter for transcription activation and oncogenesis. Nat. Cell Biol..

[26] Kasashima H (2016). Lysyl oxidase is associated with the epithelial-mesenchymal transition of gastric cancer cells in hypoxia. Gastric Cancer.

[27] El-Haibi CP (2012). Critical role for lysyl oxidase in mesenchymal stem cell-driven breast cancer malignancy. Proc. Natl. Acad. Sci. USA.

[28] Romagnoli M (2012). Epithelial-to-mesenchymal transition induced by TGF-beta1 is mediated by Blimp-1-dependent repression of BMP-5. Cancer Res..

[29] Perissi V, Aggarwal A, Glass CK, Rose DW, Rosenfeld MG (2004). A corepressor/coactivator exchange complex required for transcriptional activation by nuclear receptors and other regulated transcription factors. Cell.

[30] Dontu G (2003). In vitro propagation and transcriptional profiling of human mammary stem/progenitor cells. Genes Dev..

[31] Prat A (2010). Phenotypic and molecular characterization of the claudin-low intrinsic subtype of breast cancer. Breast Cancer Res..

[32] Ramadoss S, Li J, Ding X, Al Hezaimi K, Wang CY (2011). Transducin beta-like protein 1 recruits nuclear factor kappaB to the target gene promoter for transcriptional activation. Mol. Cell Biol..

[33] Postigo AA (2003). Opposing functions of ZEB proteins in the regulation of the TGFbeta/BMP signaling pathway. EMBO J..

[34] Postigo AA, Depp JL, Taylor JJ, Kroll KL (2003). Regulation of Smad signaling through a differential recruitment of coactivators and corepressors by ZEB proteins. EMBO J..

[35] Sanchez-Tillo E (2015). ZEB1 and TCF4 reciprocally modulate their transcriptional activities to regulate Wnt target gene expression. Oncogene.

[36] Choi HK (2011). Reversible SUMOylation of TBL1-TBLR1 regulates beta-catenin-mediated Wnt signaling. Mol. Cell.

[37] Sanchez-Tillo E (2011). beta-catenin/TCF4 complex induces the epithelial-to-mesenchymal transition (EMT)-activator ZEB1 to regulate tumor invasiveness. Proc. Natl. Acad. Sci. USA.

[38] Taube JH (2010). Core epithelial-to-mesenchymal transition interactome gene-expression signature is associated with claudin-low and metaplastic breast cancer subtypes. Proc. Natl. Acad. Sci. USA.

[39] Hennessy BT (2009). Characterization of a naturally occurring breast cancer subset enriched in epithelial-to-mesenchymal transition and stem cell characteristics. Cancer Res..

[40] Tan TZ (2014). Epithelial-mesenchymal transition spectrum quantification and its efficacy in deciphering survival and drug responses of cancer patients. EMBO Mol. Med..

[41] Medvedeva YA (2015). EpiFactors: a comprehensive database of human epigenetic factors and complexes. Database (Oxf.).

[42] Mulder KW (2012). Diverse epigenetic strategies interact to control epidermal differentiation. Nat. Cell Biol..

[43] Mock K (2015). The EMT-activator ZEB1 induces bone metastasis associated genes including BMP-inhibitors. Oncotarget.

[44] Batlle E (2000). The transcription factor snail is a repressor of E-cadherin gene expression in epithelial tumour cells. Nat. Cell Biol..

[45] Yu M (2009). A developmentally regulated inducer of EMT, LBX1, contributes to breast cancer progression. Genes Dev..

[46] Ceballos-Chavez M (2012). Control of neuronal differentiation by sumoylation of BRAF35, a subunit of the LSD1-CoREST histone demethylase complex. Proc. Natl. Acad. Sci. USA.

[47] Falasca M, Raimondi C, Maffucci T (2011). Boyden chamber. Methods Mol. Biol..

[48] Ceballos-Chavez M (2015). The chromatin Remodeler CHD8 is required for activation of progesterone receptor-dependent enhancers. PLoS Genet..

[49] Ciriello G (2015). Comprehensive molecular portraits of invasive lobular breast. Cancer Cell.

