# Supplementary material for: TBL1 is required for the mesenchymal phenotype of transformed breast cancer cells
Source: Cell Death Dis. 2019 Jan 31;10(2):95. doi: 10.1038/s41419-019-1310-1 (PMC6355934; doi:10.1038/s41419-019-1310-1)
Supplement: Supplementary file 1 — Supplementary Fig S1 [file 41419_2019_1310_MOESM1_ESM.pdf]

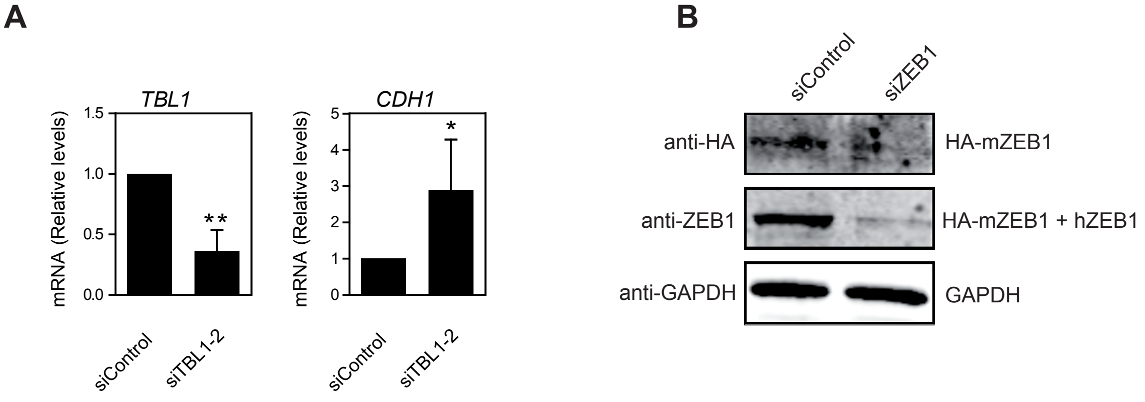

Supplementary Figure S1. a Verification of knockdown of TBL1 with a different siRNA increases CDH1 gene expression. TBL1 and CDH1 mRNA expression levels were analyzed by RT-qPCR in HMEC-RAS-ZEB1 cells at 72 hours after transfection with siTBL1-2. Data are the average of n = 6 data from three independent experiments ± SD. *, $P < 0.05$; **, $P < 0.01$ versus control by ANOVA. **b** Verification of knockdown of ZEB1. HMEC-RAS-ZEB1 cell were transfected with a siRNA that targets both the endogenous hZEB1 and the transgenic HA-mZEB1. 72 hours after transfection, protein extracts were subjected to western blotting using the antibodies indicated on the left. Identified proteins are named on the right.
