# Supplementary material for: TBL1 is required for the mesenchymal phenotype of transformed breast cancer cells
Source: Cell Death Dis. 2019 Jan 31;10(2):95. doi: 10.1038/s41419-019-1310-1 (PMC6355934; doi:10.1038/s41419-019-1310-1)
Supplement: Supplementary file 2 — Supplementary Fig S2 [file 41419_2019_1310_MOESM2_ESM.pdf]

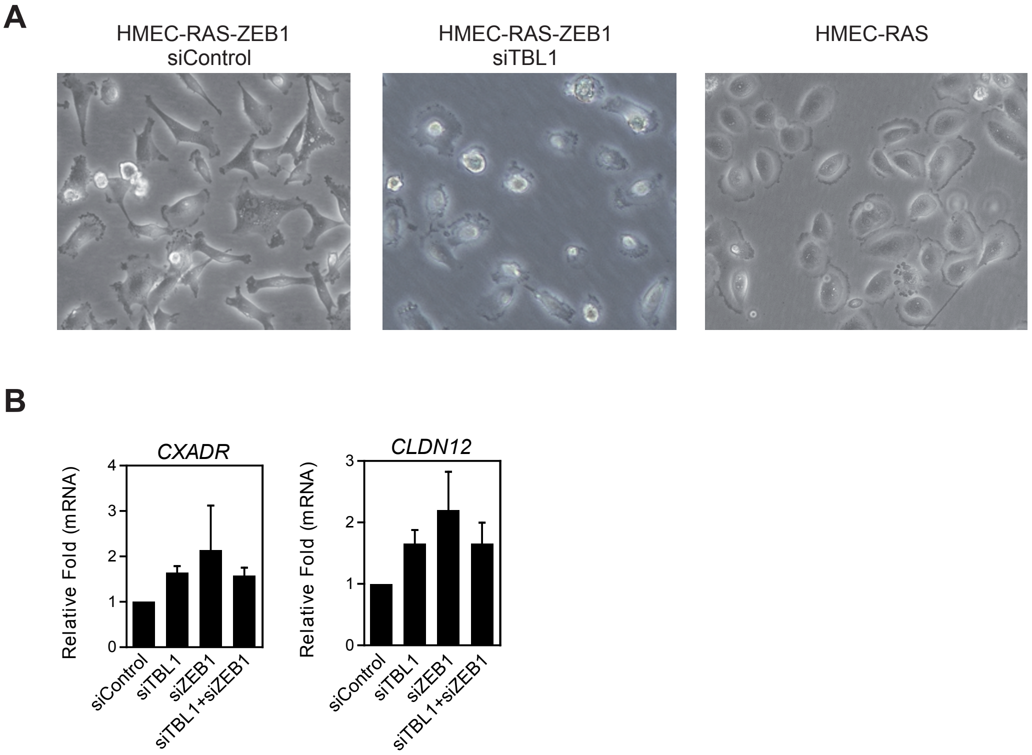

Supplementary Figure S2. a HMEC-RAS-ZEB1 cells depleted of TBL1 displayed a decreased mesenchymal-like morphology. Phase-contrast images of HMEC-RAS-ZEB1 cells transfected with the indicated siRNAs versus HMEC-RAS cells. **b** Depletion of TBL1 causes derepression of epithelial genes. *CXADR* and *CLDN12* mRNA expression levels were analyzed by RT-qPCR in HMEC-RAS-ZEB1 cells 72 hours after transfection with siTBL1, siZEB1 of both siRNAs together. Data are the average of n = 6 data from three independent experiments ± SD.
