# Supplementary material for: TBL1 is required for the mesenchymal phenotype of transformed breast cancer cells
Source: Cell Death Dis. 2019 Jan 31;10(2):95. doi: 10.1038/s41419-019-1310-1 (PMC6355934; doi:10.1038/s41419-019-1310-1)
Supplement: Supplementary file 3 — Supplementary Fig S3 [file 41419_2019_1310_MOESM3_ESM.pdf]

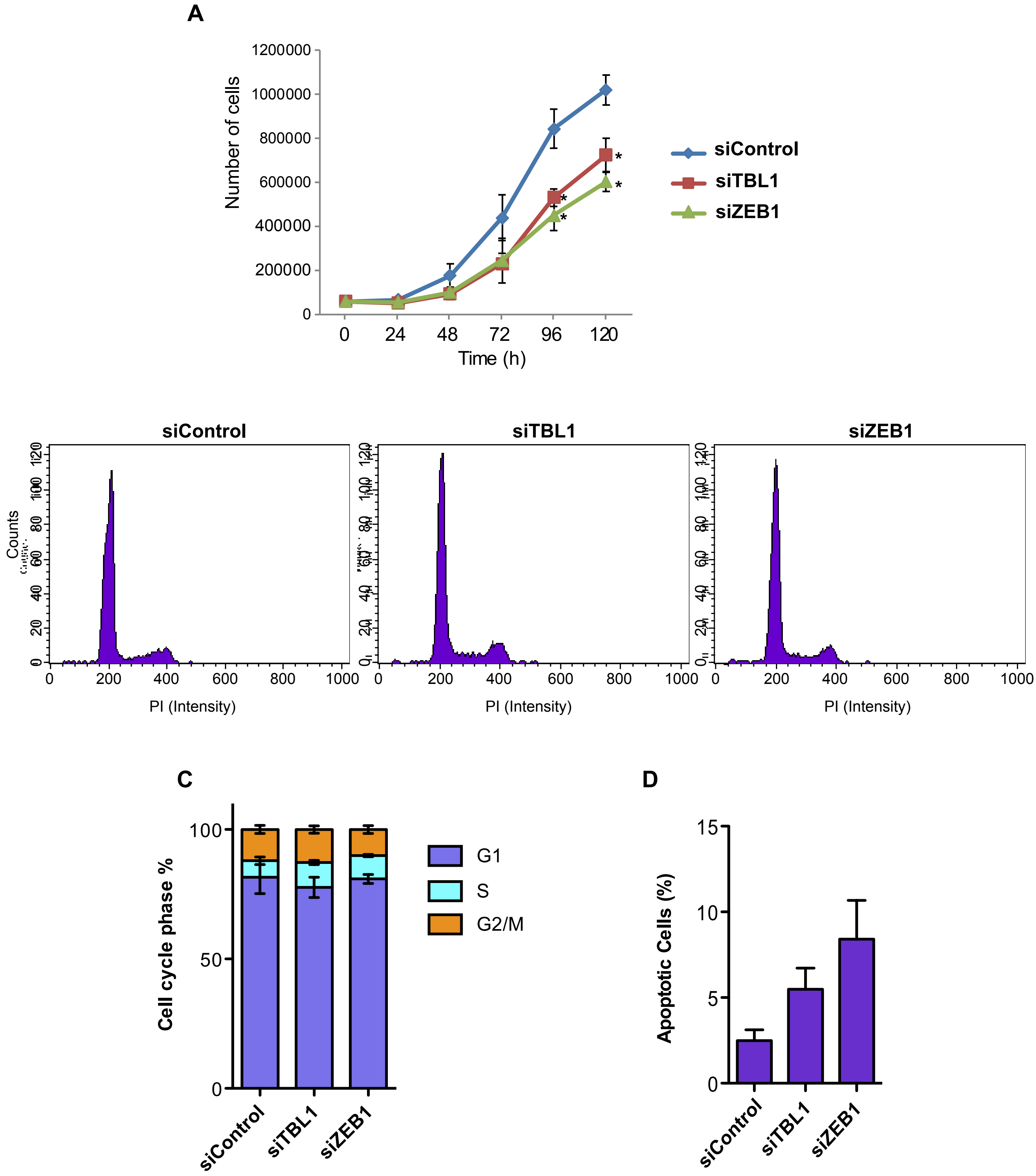

Supplementary Figure S3. a Growth curve of HMEC-RAS-ZEB1 cells treated with the indicated siRNA. **b** Flow cytometry profile of HMEC-RAS-ZEB1 cells, 72 hours after transfection of the indicated siRNAs. Representative experiments are shown. **c** Quantification of cell cycle phases from three independent flow cytometry experiments. **d** Level of apoptosis was determined 72 hours after transfection of the indicated siRNA, by measuring the percentage of cells containing a subG1 DNA content by flow cytometry. **a, c, d**. Data are the average of three independent experiments. Error bars represent standard deviation. Significance respect to the siControl was tested by using Student's t-test. * $P < 0.05$; ** $P < 0.01$.
