# Supplementary material for: TBL1 is required for the mesenchymal phenotype of transformed breast cancer cells
Source: Cell Death Dis. 2019 Jan 31;10(2):95. doi: 10.1038/s41419-019-1310-1 (PMC6355934; doi:10.1038/s41419-019-1310-1)
Supplement: Supplementary file 5 — Supplementary Table S2 [file 41419_2019_1310_MOESM5_ESM.doc]

**Table S2. siRNAs sequences**

| Gene | siRNA name | siRNA sequence |
| --- | --- | --- |
| *TBL1* | siTBL1 | CCACACAGGAGAAGCCAAA |
| *TBL1* | siTBL1-2 | GGAAAUAGAUGGAGAGGUU |
| *ZEB1* | siZEB1 | GAUGCAAGCUGGACAGAUU |
