# Supplementary material for: TBL1 is required for the mesenchymal phenotype of transformed breast cancer cells
Source: Cell Death Dis. 2019 Jan 31;10(2):95. doi: 10.1038/s41419-019-1310-1 (PMC6355934; doi:10.1038/s41419-019-1310-1)
Supplement: Supplementary file 6 — Supplementary Table S3 [file 41419_2019_1310_MOESM6_ESM.doc]

**Table S3. Primers used for RT-qPCR and ChIP-qPCR**

| Gene | Forward primer sequence | Reverse primer sequence |
| --- | --- | --- |
| Primers used for RT-qPCR | | |
| *TBL1* | CACACAGGAGAAGCCAAACA | GCCTGCACACATGGATACAC |
| *GAPDH* | GAGTCAACGGATTTGGTCGT | AATGAAGGGGTCATTGATGG |
| *hZEB1* | AGGCAGATGAAGCAGGATGT | GCCTCAGGAAAAATGACAGC |
| *CDH1* | CGACCCAACCAAGAATCTA | ACCCACCTCTAAGGCCATCT |
| *mZEB1* | GCCAGCAGTCATGATGAAAA | TATCACAATACGGGCAGGTG |
| *CLDN12* | TTGAGCCCTCATCAAGCTCT | CTCTCCCATGGCTGGATAAA |
| *CXADR* | TAAGCCTTCAGGTGCGAGAT | CCATGAAGTGGGCATTTTCT |
| Primers used for ChIP-qPCR | | |
| *CDH1* | GGCCGGCAGGTGAAC | GGGCTGGAGTCTGAACTGAC |
| *ZEB1* | TGGCCTGTGGATACCTTAGC | CACACGGTGCTTGTCTCACT |
